# *In vitro* toxicity assessment and enhanced drug solubility profile of green deep eutectic solvent derivatives (DESDs) combined with theoretical validation

**DOI:** 10.1039/c9ra10320a

**Published:** 2020-06-24

**Authors:** Anil Kumar Jangir, Bhoomi Lad, Unnati Dani, Nehal Shah, Ketan Kuperkar

**Affiliations:** Department of Applied Chemistry, Sardar Vallabhbhai National Institute of Technology Surat-395 007 Gujarat India ketankuperkar@gmail.com; Department of Biotechnology, Shree Ramkrishna Institute of Computer Education and Applied Science Surat-395 001 Gujarat India; Department of Chemistry, Bhagwan Mahavir College of Science and Technology Surat-395 017 Gujarat India

## Abstract

Green solvents are actively taking over as the absolute replacement of intrinsic toxic volatile organic solvents. This is conspicuously analyzed in this study, which mentions the preparation of green deep eutectic solvent derivatives (DESDs) composed of choline chloride (ChCl) as the hydrogen bond acceptor (HBA) and two acids, *viz.*, oxalic acid (OX) and citric acid (CA) as preliminary hydrogen bond donors (HBDs) with ethylene glycol (EG) and glycerol (GLY) as secondary HBDs in an equimolar ratio. This study exposes the vigilant choice of the type and mole ratio of HBA and HBDs, which permit the extended stability of the formulated DESDs in the liquid state even below the room temperature. The prepared DESDs were well-characterized by FT-IR spectroscopy. Furthermore, this work aimed at investigating their antimicrobial activity towards selected bacterial and fungal strains expressed in terms of viscosity measurements. The *in vitro* toxicity profiles in terms of cytotoxicity (human cervical cancer cell line) and genotoxicity (DNA fragmentation), which have not been reported to date, were also assessed for the prepared DESDs. Tuning the HBA and HBDs in selected DESDs for promising biological activity was found to have ethical implications. In addition, this study focused on the solubilization enhancement of the local anaesthetic drug lidocaine (LDC) in the stated DESDs as a function of water composition, and higher solubility was observed due to the fair intermolecular hydrogen bonding interactions between LDC and DESDs, which was further validated using the computational simulation approach. In addition, the electron-donating and accepting sites were depicted by 3D-molecular electrostatic potential (3D-MEP) for the examined systems. The observed variations were attributed to the changes in the solvation capacity, viscosity and ionic strength of pure DESDs as a function of water concentration. Finally, this study supports the role of dual HBDs in leading to the formation of stable DESDs with noteworthy action towards drug solubilization and a remarkable biological response.

## Abstract 2

Scheme illustrating the sustainable preparation of deep eutectic solvent derivatives (DESDs), their biological response and water-insoluble hydrophobic drug dissolution trend.

## Graphical abstract

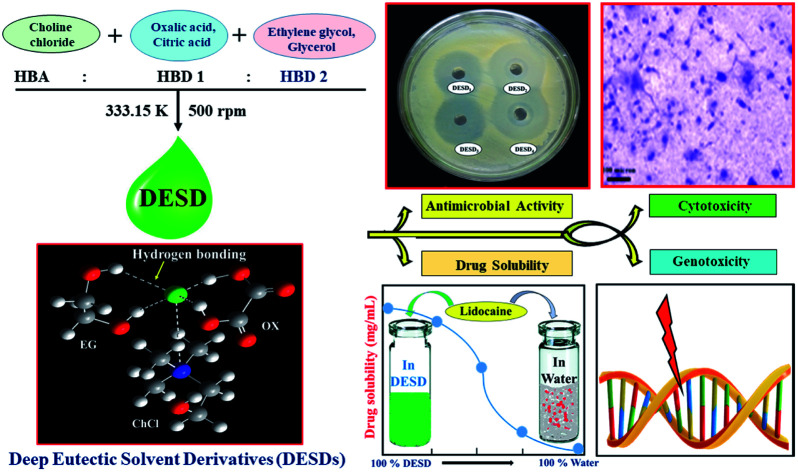

## Introduction

Deep eutectic solvents (DESs) are addressed as low-melting mixtures (LMMs), deep eutectic ionic liquids (DEILs), and low-transition temperature mixtures (LTTMs).^1,2^ Generally, DESs refer to liquids that have a eutectic temperature at a particular composition. They can be potential substitutes to ionic liquids (ILs) as they are inflammable and possess low volatility, low melting point and vapor pressure, and elevated chemical and thermal stability with high solubility analogous to that of ILs.^3,4^ Studies have reported a large number of DES formulations comprising mixtures of ammonium- or phosphonium-based salts as hydrogen bond acceptors (HBAs) with a diversified class of hydrogen bond donors (HBDs), such as amides, glycols, and carboxylic acids, that present a strong depression in the melting point at a specific molar ratio; this can be due to a self-association mechanism, *i.e.*, charge delocalization *via* hydrogen bonding between the individual HBA and HBD counterparts, thereby making DESs more homogeneous, transparent and very stable at room temperature.^5–8^ These DESs display spectacular physiognomies unified with outstanding biocompatibility, which compel the production of eutectic mixtures with several HBDs. Being addressed as designer solvents, the characteristics of DESs can be tailor-made merely by choosing suitable combinations of HBA and HBDs in varied molar ratios. These can be of natural origin and/or non-hazardous and are widely acknowledged as a fresh generation of green solvents. Indeed, DESs are thus exploited promisingly in a range of applications, including extraction, carbon dioxide capture, biocatalysis, electrochemistry, and biomedical applications due to their feasible synthesis process using cheap starting materials.^1,6–9^ Notwithstanding these accomplishments, it is indispensable to assess the safety and health and environmental impacts of these DESs and address them satisfactorily.

In addition, DESs composed of single HBDs (binary DESs) have been reported to exhibit lower or moderate toxicity towards microorganisms due to their poor stability at room temperature, while DESs composed of dual HBDs (ternary DESs), also known as deep eutectic solvent derivatives (DESDs), are more toxic due to the delocalised charges of hydrogen bonding, and measuring their eutectic point is difficult.^10–14^ In fact, the wide range of possibilities and wide adaptability offered by these solvents to the pharmaceutical industry, as well as the biological sector/biomaterial science, are yet to be unfolded, and secure mixtures must be created since their environmental footprint *i.e.*, their toxicity data are not yet systematically explored.^15,16^ Recently, researchers have focused on investigating the toxicity profiles of phosphonium- and choline chloride-based DESs containing glycerine, ethylene glycol, triethylene glycol, and urea as HBDs on varied cell lines and microorganisms, and among the selected HBDs, ethylene glycol exhibited the best response.^17,18^ Thus, cytotoxicity and genotoxicity research in this direction was our intent, which may prove obligatory to develop a better understanding of these DESDs and serve as a basis for the better selection of chemicals with the potential to disrupt abnormal human and animal cells. Correspondingly, the antimicrobial impression of these DESDs was explored.

Furthermore, another important characteristic property that needs to be explored for pharmaceutical formulations and manufacturing design is drug solubility. One of the major problems with the current active pharmaceutical ingredients (APIs) is their low water-solubility, resulting in low bioavailability and permeability. Researchers have reported that imidazolium-based ILs provide enormous varieties with known cations and anions for the development of a particular solvent for focused APIs. The fact that ILs have tremendous physical properties, certain toxicity problems, unfavorable biodegradability along with high cost and environmental implications has compelled the recent scientific research scenario to anticipate DESs as proficient substitutes to ILs.^19–21^ Therefore, the need to develop a new category of solvents with no toxicity and biodegradability has been looked upon as a way to resolve the issue of API solubility. Researchers have reported several pharmaceutical eutectic mixtures composed of menthol/testosterone, ibuprofen/menthol and lidocaine/procaine. However, these eutectic mixtures of local anesthetics (EMLAs) seemed to be unsuitable in terms of uptake, aqueous solubility, and thermal prosperity.^22^ However, the innovation of therapeutic DESs acquired from naturally occurring chemicals (DESDs) is a shove in this field of research, which can comprehensively enhance the API solubility enhancement.^14,23–25^ In this direction, only a few theoretical analyses using molecular dynamics and the functional theory have markedly demonstrated the solubility aspect of the drug at the microscopic level to gain deep insights about the intermolecular interactions in DESs in terms of the type of HBA/HBD.^26,27^

In the context mentioned above, this work offers insights into the preparation of non-toxic green DESDs using ammonium-based choline chloride (ChCl) as the HBA combined with oxalic acid (OX)/citric acid (CA) and ethylene glycol (EG)/glycerol (GLY) as the dual HBDs in a favourable molar ratio for a stable DESD formulation. The spectral and viscosity studies emphasized the vigilant selection of HBA and HBDs, depicting favorable and extensive intermolecular hydrogen bonding interactions. Furthermore, the understanding of the *in vitro* potentials of these DESDs could be rationalized in terms of its biocompatibility. Additionally, the solubility test of the local anaesthetic hydrophobic drug lidocaine (LDC) in the aforementioned DESDs was performed as a function of water composition to accomplish a deep understanding of solubility enhancement due to fair intermolecular hydrogen bonding interactions between LDC and DESDs. The observed variations were attributed to the changes in the solvation capacity, viscosity and ionic strength of pure DESDs as a function of water concentration. Additionally, this study presents the theoretical (computational) validation of LDC solubility in the studied DESDs.

## Experimental details

### Materials

**Scheme 1:**
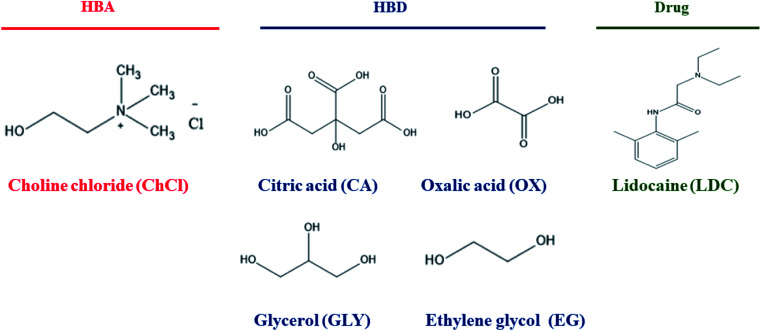
Chemical ingredients employed in this study with their respective structures.

HBA: choline chloride (ChCl; purity > 99%) and HBDs: oxalic acid (OA; purity > 99%), citric acid (CA; purity > 99%), glycerol (GLY; purity > 99%), and ethylene glycol (EG; purity > 99%) were procured commercially from S. D. Fine Chem. Ltd., India. Lidocaine (LDC) (solubility in water ∼ 3 mg mL^−1^) was procured from MP Biomedicals, France. The structures of the selected chemicals used in this study are presented in Scheme 1. All the compounds used for this study were of analytical grade. These ingredients were kept in a vacuum chamber to avoid water gain in the samples and used as such without further purification (except ChCl, which was dried in vacuum at 383.15 K for 24 h before use).

### Media

Stock cultures of the Gram-positive (*Escherichia coli*: ATCC-23564) and the Gram-negative (*Staphylococcus aureus*: ATCC-9144) bacteria and the fungus (*Candida albicans*: ATCC-10231) were purchased from National Chemical Laboratory, Pune, India and used as received. Mueller–Hinton (Hi-Media, M173, pH – 7.3 ± 0.1) agar was used as the culture medium in this study. The HeLa cell line (human cervical cancerous cell line) used in this work was acquired from the National Centre for Cell Sciences (NCCS), India.^28^

### Methods

#### Preparation of DESDs

In this study, a sustainable heating route was employed for the preparation of the ChCl-based DESs. Four DESDs were prepared as a series with following the combinations of HBA : HBD1 : HBD2–ChCl : OX : EG (DESD_1_), ChCl : OX : GLY (DESD_2_), ChCl : CA : EG (DESD_3_), and ChCl : CA : GLY (DESD_4_) at varying molar ratios (1 : 1 : 0.25, 1 : 1 : 0.50, 1 : 1 : 0.75 and 1 : 1 : 1) using a high precision (1 × 10^−4^ g) analytical balance (Model: AW 220, GR220, Shimadzu, Japan) with the minimum uncertainty of less than 2 × 10^−4^ g in weight fractions.^29*a*^ All the ingredients were heated in screw-capped glass vials at 333.15 K with constant stirring at around 500 rpm until a clear and homogeneous liquid was formed. To reduce the content of water/moisture, the prepared DESDs were further kept in a vacuum oven at 333.15 K for 24 h. Later, the prepared DESDs were stored in wrapped vials for further utilization. The prepared DESD sample with the ratio of 1 : 1 : 1 was found to be more suitable as it had the appropriate thermodynamic stability and versatile fluidity. As no glass transition temperature was observed up to 273.15 K for the prepared DESDs, DSC was not needed and hence not performed.

## Characterization

### Fourier transform infrared spectroscopy (FT-IR)

The prepared DESDs were characterized using a DRS (Model: 8400-S-Shimadzu) FT-IR spectrometer with a 2 cm^−1^ resolution and 45 scans in the range 4000–400 cm^−1^ by employing a NaCl optical window at room temperature.^29*a*^

### Kinematic viscosity (*ν*)

The kinematic viscosities (*ν*) for all the four prepared DESDs were scanned using a well-standardized Cannon Ubbelohde viscometer suspended vertically in a water bath (Model: 14 L-SS, Equiptron, India). A digital electronic stopwatch with a resolution of 0.01 s was used to measure the travel time (in seconds) of the sample. A minimum of three measurements was performed for each sample, and their average value was considered for the calculation of kinematic viscosity (*ν*).^29*a*^ The uncertainty in *ν* and temperature were estimated to be ± 0.004 cSt and ± 0.02 K, respectively.

### Biocidal assay

The Kirby Bauer method was employed to evaluate the antimicrobial performance *i.e.*, the toxicity of the DESDs against the tested microorganisms.^28^

### Cytotoxicity assay

The proliferation of HeLa cells was tested in the presence of the prepared DESDs according to a protocol reported in the literature.^28,29*b*^

### Genotoxicity test

For the DNA fragmentation study, the HeLa cells were cultivated and incubated for 24 h (same procedure as in the cytotoxicity test). After seeding the cells, the culture plates were treated with the DESDs (at two different concentrations ∼50 μL and ∼200 μL). Fluorouracil and mitomycin C (∼100 μL) were used as positive controls. After extraction, ∼20 μL DNA from each sample along with ethidium bromide stain was loaded in a gel, and UV light was used to visualize the plate. DNA from the control and DESD-treated HeLa cells was assessed to understand whether solvents have any effect on the integrity of DNA. Here, the agarose gel electrophoresis findings displayed a smear for the treated group.^17^

### Drug solubility

The most popular shake-flask method was employed for the drug solubility experiment. Here, ∼25 mg of LDC was solubilized in 1 mL of each DESD by stirring at room temperature and then, placed in a temperature-controlled water bath for 2 days to attain equilibrium. Later, the sample solutions were filtered (0.45 μm, Millipore, MA) before they attained the maximum solubility of LDC in DESDs. The clear solutions were then assayed using a double beam spectrophotometer (Model: Shimadzu, Japan) to measure the absorbance spectra, which depict drug solubilization.^30^

### Computational study

The HOMO–LUMO frontier orbital compositions were obtained at the theoretical level using the semi-empirical method with the basis set 3-21 G *via* the open-source code: Gaussian 09W calculation window using the Gauss View 5.0.9 software package.^28*a*,29*a*^

## Results and discussion

### Physical appearance

**Fig. 1:**
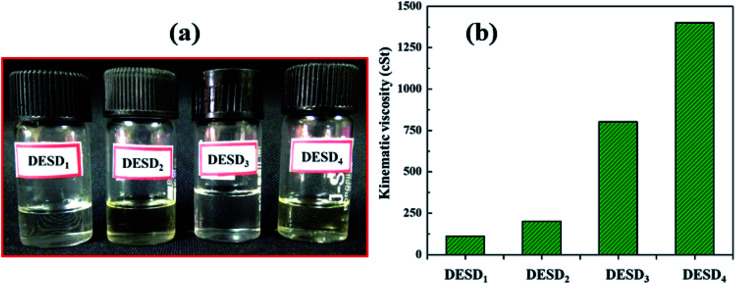
(a) Schematic of the various DESDs illustrating stable physical appearance and (b) kinematic viscosity (*ν*).

The ChCl-based binary DESs with only primary HBDs (CA and OX) are not stable in the liquid state at room temperature as they have high freezing points, which is a significant barrier for their smooth utilization.^3^ Consequently, EG and GLY in varying degrees of polarity were selected as the secondary HBDs in anticipation that the assimilation of additional foreigner HBDs will decrease the freezing point of these DESs, resulting in a DESD and making the system more stable at room temperature. Fig. 1 illustrates the four prepared DESDs, which appeared very clear and transparent with no insoluble particles. These DESDs did not show any appreciable phase changes up to 273.15 K, which confirmed their extended and enhanced stability.

Studies have reported the influence of HBDs on the viscosity of DESs.^29*a*,33,34*a*^ The viscosity values of the less viscous DESD_1_ and DESD_2_ were ∼110.521 cSt and ∼200.412 cSt, respectively, while those of the more viscous DESD_3_ and DESD_4_ were around ∼800.451 cSt and ∼1400.221 cSt, respectively. Such drastic variation in the DESD viscosity trend was attributed to the hydrogen bonding and van der Waals interactions, which were further verified by spectral studies.

### Spectral validation

FT-IR is a compelling analytical technique that can provide ample details on the chemical structures based on the functional groups present in the prepared DESDs.

**Fig. 2:**
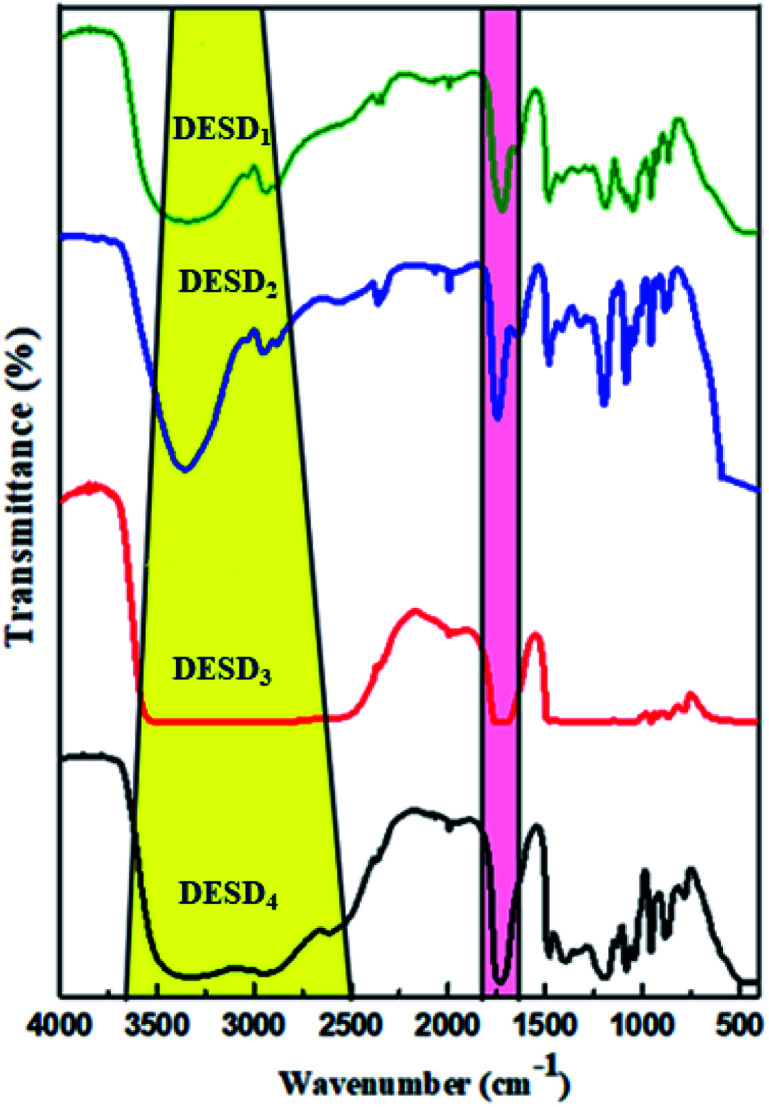
FT-IR spectra inferring the correct preparation of the four DESDs.

As illustrated in Fig. 2, all the DESDs showed comparable functional spectra because the precursors chosen as the dual HBDs for DESD preparation had almost similar functional groups. The presence of hydrogen bonding (region highlighted in yellow) was dominantly apparent in the IR spectra of all the prepared DESDs as a broad vibrational band of the –OH group around ∼3700–3100 cm^−1^, which confirmed the strong hydrogen bonding and matched well with a previously reported work.^31^ However, these bands are affected by the additive strength *i.e.*, hydrogen bonding between HBA and HBDs, resulting in an increase or decrease in the breadth of the band. In our case, a close perusal of the DESD spectral stack revealed wider bands for DESD_3_ and DESD_4_, which depicted greater hydrogen bonding interactions between the HBA and HBDs. This trend could be attributed to the hydrogen bond networks leading to electron transfers that involve hydrogen atoms, thereby decreasing the force constant. However, the peaks at ∼2942 cm^−1^ in the DESD_1_ spectrum and ∼2955 cm^−1^ in the DESD_2_ spectrum matched the vibrational bands of alkyl groups, whereas the same band was observed to overlap in DESD_3_ and DESD_4_ due to the extensive hydrogen bonding. In addition, the peak at ∼1700 cm^−1^ (region highlighted in pink color) could be ascribed to the vibrational frequency of aliphatic carbonyl groups present in the acid, which confirmed the involvement of the selected acids in DESD preparation.^34*b*^ Moreover, the peak around ∼1480 cm^−1^ was designated to the bending mode of the alkyl groups present.

### Antimicrobial action

The toxicity profile of a DES depicts the dependency on its concentration, the nature of individual compounds, solubility, osmolality, pH and the type of bacterial cell membrane.^17*b*,34*a*^ However, the behavior of DESs expressed in terms of viscosity is considered very vital in studying the intercellular activities at the cellular level. The viscosity of the solvent not only affects diffusion within the biological systems, but also has roles in vital processes, *viz.*, protein–protein interactions, transportation of small solutes/macromolecules, and signal-transduction in living cells.^34^ Studies have further specified that despite resulting in high viscosities, DESs prepared from sugars^15,34*a*^ are relatively less toxic to cells than those composed of organic acids.^35,37^ It is the cholinium (Ch^+^) cations of DESs, which partly dissociate in aqueous solutions, that interact with the charged groups on the surface of the membrane, leading to its disruption.^20,35,36^ Further, ternary DESs or DESDs exhibit more toxicity due to the delocalised charges of the hydrogen bonds in comparison with their individual components. Recently, research has revealed the enhanced absorption of DESs in the lipid bilayer of animal cells, while their consequence on the microbial membranes is still indefinite.^10–13,17,34*c*^

**Fig. 3:**
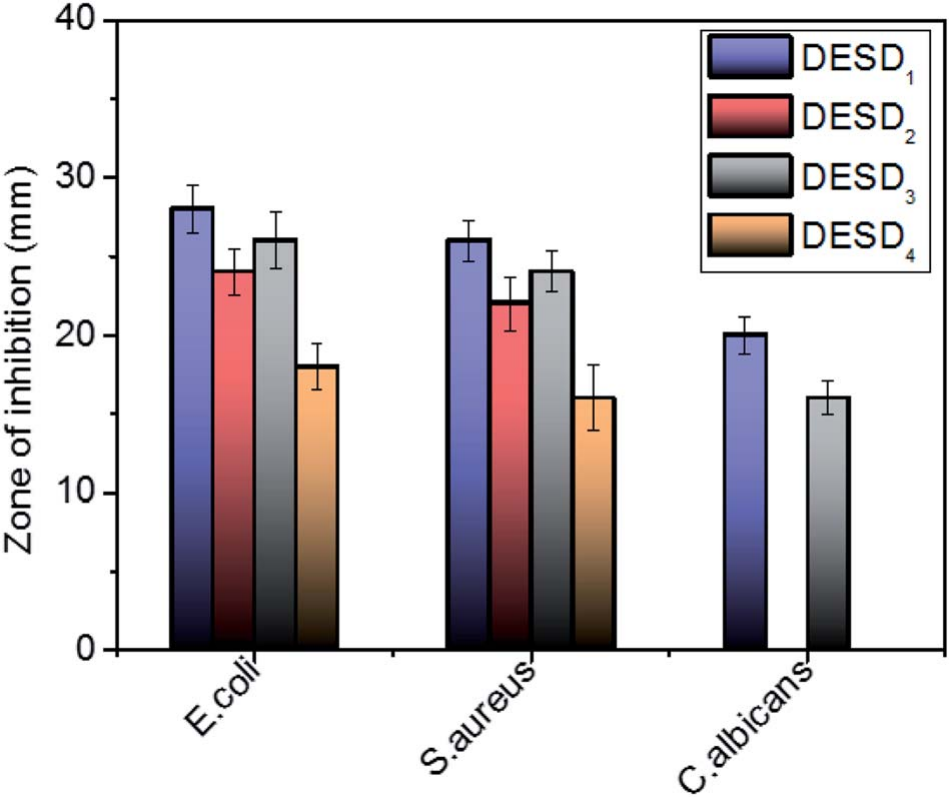
Graphical representation of the antimicrobial activity of the DESDs expressed in terms of the zone of inhibition (mm) (data are presented as mean ± standard error).

The bacterial cell wall that possesses peptidoglycans gives firmness and strength to the cell membrane, while the fungus cell wall is more rigid compared with that of bacteria. The *in vitro* antimicrobial activity assay of the prepared DESDs towards selected pathogenic organisms showed differences in the inhibitory activity (Fig. 3). DESD_1_ showed the greatest inhibition efficiency than the other DESDs for the selected microbes. In particular, DESD_1_ showed the best activity results, and DESD_3_ showed moderate performance on the bacteria and fungus, whereas DESD_2_ and DESD_4_ did not show any action on the fungus. The best plausible explanation for this could be the fair fluidic nature of the DESD media containing EG, which imparts an ideal viscosity compared with the high viscosity of the GLY-containing DESDs. Moreover, the DESD_2_ and DESD_4_ systems are highly acidic in nature, which facilitates the penetration of the lipid layer, resulting in high toxicity against bacteria and poor performance against the fungus.

### Cytotoxicity

The human cervical cancer cells assessed in this work showed a reduction in cell viability because of the high inhibitory effect of DESDs. This can be explained by the fact the cancerous cells uptake more DESDs and easily incorporate them within, thereby causing a severe effect.^38–40^ Studies have also perceived that the variety and mole ratio of HBD have a potent effect on the cytotoxicity and proliferation rate than their individual components, indicating the synergistic effect of the mixtures. The cytotoxic profiles of several ChCl-based DESDs have been reported on fibroblast cells, and their toxic effect has been correlated and expressed in terms of their viscosity.^41^

**Fig. 4:**
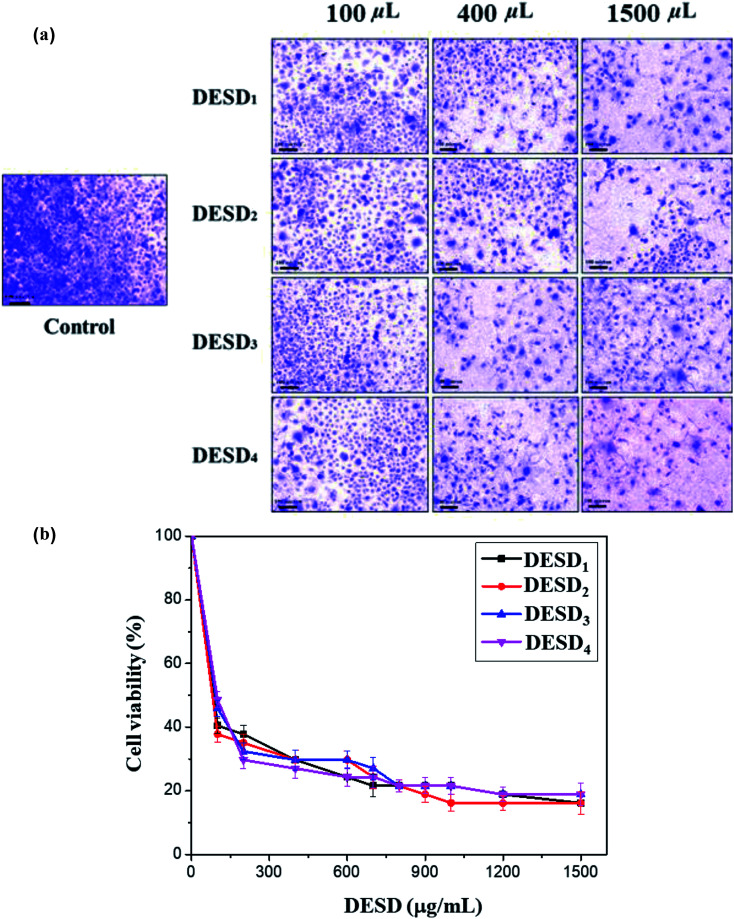
(a) Fluorescence micrographs of human cervical cancer cells after dose-dependent treatment with the DESDs. (b) The inhibitory concentrations (IC_50_ μg mL^−1^) derived from the cell viability–concentration curves (data are presented as mean ± SE).

In this assay, the prepared DESDs exhibited higher toxicity towards the HeLa cell line compared with those in the previous study due to the presence of secondary HBDs, namely EG and GLY. The highest inhibitory effect was observed for DESD_1_, while the lowest effect was observed for DESD_4_ against the cancerous cells (Fig. 4a).

For better evaluation, the IC_50_ values were derived from the dose–response graphs, where the DESDs exhibited low IC_50_ values, thus suggesting them to be very effective towards the selected cells (Fig. 4b). DESD_1_ showed an IC_50_ value of ∼150 μg mL^−1^, while DESD_4_ had the IC_50_ value ∼200 μg mL^−1^. The IC_50_ trend was found to increase in the order: DESD_1_ > DESD_3_ > DESD_2_ > DESD_4_. These results elucidated the proliferation activity and correlated with the composition (the type of HBDs) and the concentration of the DESDs.

### Genotoxicity

DNA damage is one of the common responses towards various stress conditions. Different methods have been developed to accurately evaluate DNA damage and fragmentation in cells. A study depicting the toxicity of DESDs has not been presented yet and thus, such an investigation can set a benchmark for the future development of DESDs.^17^

**Fig. 5:**
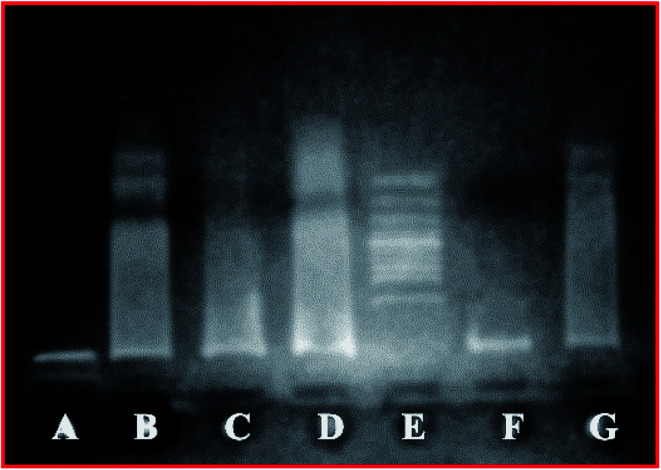
Electrophoretic analysis demonstrating the DNA fragmentation analysis of HeLa cells treated with the DESDs (as a function of concentration): (A) negative control; (B) positive control; (C) DESD_1_ ∼ 50 μL, (D) DESD_1_ ∼ 200 μL; (E) 100 bps ladder; (F) DESD_4_ ∼ 50 μL, and (G) DESD_4_ ∼ 200 μL.

The DNA fragmentation activity was examined after exposure of HeLa cells to different DESD concentrations (∼50 μL and ∼200 μL) (Fig. 5). DESD_4_ showed DNA damage at ∼50 μL (lane C), while at ∼200 μL (lane D), the destruction was greater. Low DESD_1_ exposure resulted in less DNA fragmentation in lane F (∼50 μL) than that in lane G (∼200 μL), which indicated that DNA impairment increased with the amount of solvent. In comparison, DESD_1_ was more genotoxically lethal in nature than DESD_4_.

### Drug solubility

**Fig. 6:**
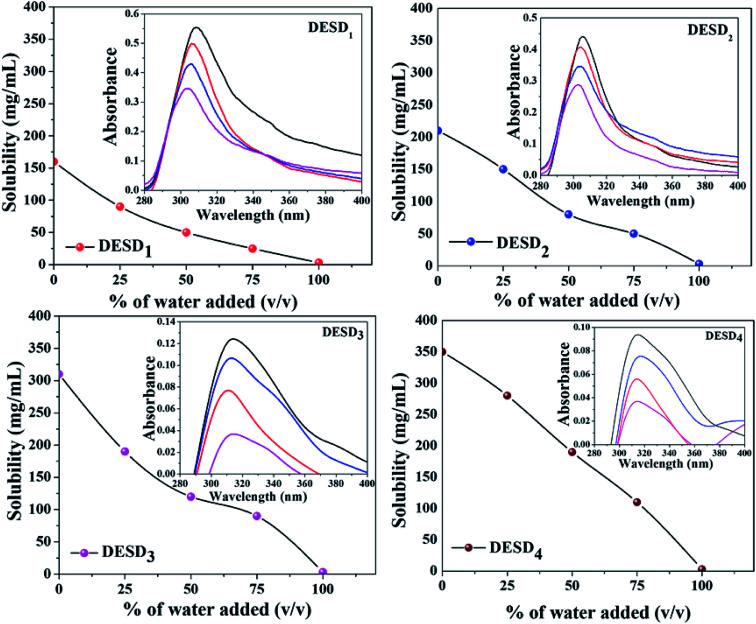
Solubility behavior of LDC as a function of DESD-water composition (insight images depict the absorbance profile with respect to the wavelength where each colored spectrum correspond to the 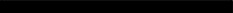 pure DESD; 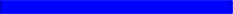 25% water + 75% DESD; 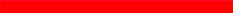 50% water + 50% DESD; and 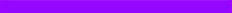 75% water + 25% DESD).

As the solvents are to be used for drug solubility, the components used for the synthesis of DESs should be non-toxic, *i.e.*, the HBA and HBD must be biocompatible and less toxic.^10,42^ Previous studies have reported the selection of HBD to be very crucial to enhance the stability and drug solubility of the DESs.^14,30^ Additionally, water can simply modify the formed hydrogen bonds in DESDs to accomplish a certain level and in this way, the dissolving capacity of the DESDs can be tailor-made.^9^ For our study, the water solubility issue of LDC was addressed in the environment of the prepared DESDs. Fig. 6 depicts that 0–100 volume% of water in the DESD–water mixtures were utilized to dissolve LDC. Moreover, it is interesting to note that the accumulation of water in all the prepared DESDs reduced solvation, which was reflected by the downtrends of the LDC solubility profiles of the different DESDs. The examined DESDs showed varied trends for LDC solubility, which followed the order: DESD_4_ (∼350 ± 2.0 mg mL^−1^) > DESD_3_ (∼310 ± 2.0 mg mL^−1^) > DESD_2_ (∼210 ± 2.0 mg mL^−1^) > DESD_1_ (∼160 ± 2.0 mg mL^−1^) (Fig. 6). The plausible behavior of DESD_4_ could be due to its considerably high viscosity and extended hydrogen networking, which facilitated more dissolution/encapsulation of LDC within the DESs network. LDC solubility in the prepared DESDs was comparably higher than the data for DESs reported by Li *et al.*^14^ Thus, based on the spectral results of these solvent media, it is anticipated that the solubility of LDC would be high manifold times compared with that in water, and such behavior would make the non-aqueous liquid DESDs ideal entities for drug administration. A close perusal of Fig. 6 showed that the DESD_1_ system exhibited a continuous decrease in the solubility of LDC with increasing water concentration.

The spectroscopic absorption technique was employed to probe the established interactions between LDC and DESDs. It has been noticed that the aqueous DESDs practically do not have an absorption band within the ∼300–600 nm wavelength range.^32^ Thus, based on the inset figures, the absorption bands at around ∼302 nm and ∼313 nm depicted the presence of the LDC molecule. DESD_1_ and DESD_2_ showed absorption peaks around ∼302 nm for 25% DESDs + 75% water, which further shifted to ∼305 nm besides an increase in intensity with increasing DESD concentration. On the other hand, DESD_3_ and DESD_4_ showed absorption peaks around ∼313 nm for 25% DESDs + 75% water. DESD_2_ and DESD_3_ exhibited nearly similar LDC solubility profiles, while the DESD_4_ system showed a relatively slow downtrend for LDC solubility as a function of water concentration probably due to the changes in viscosity and polarity at a certain level in the presence of water, which is directly associated with the hydrogen bonding within the DESD systems that reduces the solubility of the hydrophobic LDC in the DESDs. A similar trend in LDC solubility was observed for the other two DESD–water compositions. Such vivid behavior of LDC solubilization in the DESDs, a significant finding in this study, was further validated using a theoretical approach.

### Computational study

Studies published earlier have highlighted the simulation approach to be a very useful method that can offer insights into the molecular interactions between the examined DES and additives.^26,29*a*,45^ The computational data of the frontier molecular orbital (FMO) energies, *i.e.*, the highest occupied molecular orbital (*E*_HOMO_) and the lowest unoccupied molecular orbital energy (*E*_LUMO_) were assessed since they can be used to predict charge transfer within the molecules. Additionally, the stability of the complex/compound is supposedly based on their HOMO–LUMO band gap (Δ*E*). It is well-reported that a low value of Δ*E* refers to the potential of the electron to move from a filled orbital to a vacant orbital and thus, provides a crucial basis for evaluating the intermolecular interactions, quantity and stability of the compounds.^26,27,43–45^ The transfer of electron density from a lower orbital to a nearby (least) unoccupied orbital reflects localized density on the stable complex.

**Fig. 7:**
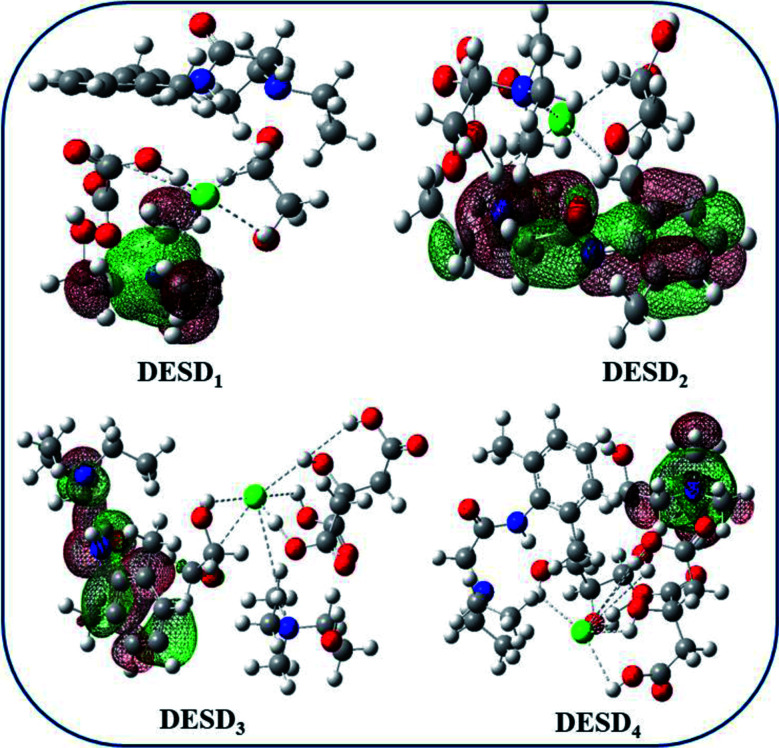
Optimized molecular structures of DESDs + LDC depicting the HOMO and LUMO lobes in red and green, respectively. The quaternary nitrogen atom is shown in blue, oxygen is shown in red colour, chlorine is shown in green colour, carbon is shown in grey and the hydrogen atoms are shown in white.

Fig. 7 depicts the fragmental distribution of the studied FMOs of the DESD + LDC systems. Table 1 summarizes the optimized parameters for pure LDC and DESDs, as well as the DESD + LDC systems. An interesting outcome of this study was that the total energy was negative for all the DESDs, which suggested that the studied systems were thermodynamically stable. Further, after LDC solubilization, the solvation caused a reasonable decrease in the Δ*E*(*E*_LUMO_ − *E*_HOMO_) value of all the DESD + LDC systems, *i.e.*, Δ*E* of DESD_4_ was around ∼0.3001 eV, while that of DESD_1_ was around ∼0.3228 eV, which suggested that DESD_4_ exhibited the greatest interactions among the studied systems. They followed the order: DESD_4_ + LDC > DESD_3_ + LDC > DESD_2_ + LDC > DESD_1_ + LDC, which is similar to our experimental findings. The decrease in Δ*E* values in the former could be due to the formation of hydrogen bonding between LDC (NH site) and the anion of ChCl, as well as between the oxygen atoms of the hydroxyl groups present in the acids and the glycols of HBDs. Moreover, the favorable electrostatic interactions of LDC (CO site) with the Ch^+^ cations and the hydrogen atoms attached to the electronegative oxygen of acids and glycols HBDs were found to be in good agreement with an earlier study.^26^

**Table 1:** Frontier molecular orbital energies and optimized parameters for the examined systems evaluated by employing the 3-21(G) model

Components	Total energy (a.u.)	*E*_HOMO_ (eV)	*E*_LUMO_ (eV)	Δ*E* (eV)
LDC	−0.0721	−0.3226	−0.0137	0.3089
DESD_1_	−0.5794	−0.3631	−0.0177	0.3454
DESD_2_	−0.6561	−0.3673	−0.0187	0.3486
DESD_3_	−0.8370	−0.3738	−0.0145	0.3593
DESD_4_	−0.8375	−0.3786	−0.0135	0.3651
DESD_1_ + LDC	−0.6666	−0.3284	−0.0056	0.3228
DESD_2_ + LDC	−0.7536	−0.3304	−0.0135	0.3169
DESD_3_ + LDC	−0.9242	−0.3199	−0.0083	0.3116
DESD_4_ + LDC	−1.0209	−0.3185	−0.0184	0.3001

**Fig. 8:**
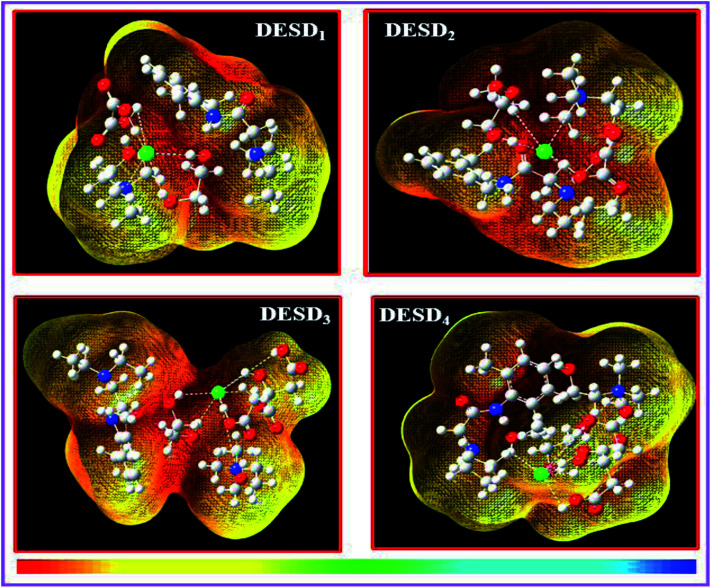
The 3D-surface plots of the molecular electrostatic potential (MEP) of the DESD + LDC complexes mapped by the linear extrapolation of values.

Orbital bond analysis has always been among the most important methods to determine the differences in electron density and charge electron-transfer within the formed complex. Fig. 8 represents the molecular electrostatic potential (MEP) with the most negative potential (assigned in red colour) representing the nucleophilic centers of the DESDs; on the other hand, the most positive potential (assigned in blue colour) represents the electrophilic site in LDC. It depicts the effective DESD–LDC interactions, which lead to better compactness and promote charge transfer within the DESD + LDC system, accompanied by a minor disruption of the HBA:HBD interaction to maintain the most relevant DESD features.

## Conclusion

The green characteristic of the designed DESDs due to their chemical diversity and a large number of possible compositions has been examined in this work for a better understanding of their biological potential and drug solubility enhancement. The prepared binary DESs comprised ChCl and CA/OX as the preliminary HBDs with modified stability that facilitated the accumulation of ethylene glycol (EG)/glycerol (GLY) as the secondary HBD in the molar ratio of 1 : 1 : 1 to form DESDs. The functional groups were effectively designated using FT-IR spectroscopy to ascertain the correct preparation of DESDs. The kinematic viscosity (*ν*) ensured that the flow behavior was suitable for biocidal activity. In sequence, their toxic effect was evaluated in terms of antimicrobial performance against selected microbial strains, and the consecutive cytotoxicity and genotoxicity strategies exhibited satisfactory outcomes towards HeLa cells. The *in vitro* approach followed the order: DESD_1_ > DESD_3_ > DESD_2_ > DESD_4_, which was more or less the reverse of the viscosity order. This study also shows an upsurge in the solubility of LDC by several orders of magnitude in the presence of DESDs than that in water and follows the order DESD_4_ > DESD_3_ > DESD_2_ > DESD_1_, which is similar to the viscosity trend. These findings revealed that the solubility can be attuned by varying the type and content (molar ratio) of HBDs. Successive studies to understand the toxicity mechanism of this particular solvent family concluded that the inherent toxicity of DESDs depended on many factors, including the molar ratio, the chemical composition of the raw materials and concentration. These findings prove that DESDs possess a promising green profile and a good prospect for wide use in the field of drug administration, which is yet under investigation.

## Funding

This research did not receive any specific grant from funding agencies in the public, commercial, or not-for-profit sectors.

## Conflicts of interest

The author declares no conflict of interest.

## Supplementary Material

## References

[cit1] Smith E. L., Abbott A. P., Ryder K. S. (2014). Chem. Rev..

[cit2] Paiva A., Craveiro R., Aroso I., Martins M., Reis R. L., Duarte A. R. C. (2014). ACS Sustainable Chem. Eng..

[cit3] Zhang Q., Vigier K. D. O., Royer S., Jérôme F. (2012). Chem. Soc. Rev..

[cit4] Kudłak B., Owczarek K., Namieśnik J. (2015). Environ. Sci. Pollut. Res..

[cit5] Abbott A. P., Capper G., Davies D. L., Rasheed R. K., Tambyrajah V. (2003). Chem. Commun..

[cit6] Carriazo D., Serrano M. C., Gutiérrez M. C., Ferrer M. L., del Monte F. (2012). Chem. Soc. Rev..

[cit7] del Monte F., Carriazo D., Serrano M. C., Gutiérrez M. C., Ferrer M. L. (2014). ChemSusChem.

[cit8] Wagle D. V., Zhao H., Baker G. A. (2014). Acc. Chem. Res..

[cit9] Morrison H. G., Sun C. C., Neervannan S. (2009). Int. J. Pharm..

[cit10] Zhao B.-Y., Xu P., Yang F.-X., Wu H., Zong M.-H., Lou W.-Y. (2015). ACS Sustainable Chem. Eng..

[cit11] Ventura S. P., e Silva F. A., Gonçalves A. M., Pereira J. L., Gonçalves F., Coutinho J. A. (2014). Ecotoxicol. Environ. Saf..

[cit12] Pernak J., Chwała P. (2003). Eur. J. Med. Chem..

[cit13] Wen Q., Chen J.-X., Tang Y.-L., Wang J., Yang Z. (2015). Chemosphere.

[cit14] Li Z., Lee P. I. (2016). Int. J. Pharm..

[cit15] Mbous Y. P., Hayyan M., Hayyan A., Wong W. F., Hashim M. A., Looi C. Y. (2017). Biotechnol. Adv..

[cit16] Fukumoto K., Yoshizawa M., Ohno H. (2005). J. Am. Chem. Soc..

[cit17] Hayyan M., Looi C. Y., Hayyan A., Wong W. F., Hashim M. A. (2015). PLoS One.

[cit18] Mbous Y. P., Hayyan M., Wong W. F., Looi C. Y., Hashim M. A. (2017). Sci. Rep..

[cit19] Chatel G., Naffrechoux E., Draye M. (2017). J. Hazard. Mater..

[cit20] Samorì D., Malferrari P., Valbonesi A., Montecavalli F., Moretti P., Galletti G., Sartor E., Tagliavini E., Fabbri E., Pasteris A. (2010). Ecotoxicol. Environ. Saf..

[cit21] Jordan A., Gathergood N. (2015). Chem. Soc. Rev..

[cit22] Wang H., Gurau G., Shamshina J., Cojocaru O. A., Janikowski J., MacFarlane D. R., Davis J. H., Rogers R. D. (2014). Chem. Sci..

[cit23] Silva J. M., Reis R. L., Paiva A., Duarte A. R. C. (2018). ACS Sustainable Chem. Eng..

[cit24] Shekaari H., Zafarani-Moattar M. T., Mokhtarpour M. (2017). Eur. J. Pharm. Sci..

[cit25] Park H. J., Prausnitz M. R. (2015). AIChE J..

[cit26] Gutiérrez A., Atilhan M., Aparicio S. (2018). Phys. Chem. Chem. Phys..

[cit27] Ley R. T., Paluch A. S. (2016). J. Chem. Phys..

[cit28] Dani U., Bahadur A., Kuperkar K. (2018). Colloid Interface Sci. Commun..

[cit29] Jangir A. K., Patel D., More R., Parmar A., Kuperkar K. (2019). J. Mol. Struct..

[cit30] Lu C., Cao J., Wang N., Su E. (2016). MedChemComm.

[cit31] Delgado-Mellado N., Larriba M., Navarro P., Rigual V., Ayuso M., García J., Rodríguez F. (2018). J. Mol. Liq..

[cit32] Pandey A., Dhingra D., Pandey S. (2017). J. Phys. Chem. B.

[cit33] Florindo A., Oliveira F. S., Rebelo L. P. N., Fernandes A. M., Marrucho I. M. (2014). ACS Sustainable Chem. Eng..

[cit34] Juneidi M. H., Ali O. M. (2016). Environ. Sci. Pollut. Res..

[cit35] Mitar A., Panić M., Prlić Kardum J., Halambek J., Sander A., Zagajski Kučan K., Radojčić Redovniković I., Radošević K. (2019). Chem. Biochem. Eng. Q..

[cit36] Zhao H., Baker G. A. (2013). J. Chem. Technol. Biotechnol..

[cit37] Bubalo M. C., Radošević K., Redovniković I. R., Halambek J., Srček V. G. (2014). Ecotoxicol. Environ. Saf..

[cit38] Radošević K., Čanak I., Panić M., Markov K., Bubalo M. C., Frece J., Srček V. G., Redovniković I. R. (2018). Environ. Sci. Pollut. Res..

[cit39] McClean R., MacCallum C., Blyde D., Holt W. V., Johnston S. D. (2006). Reprod., Fertil. Dev..

[cit40] Davis P., Bramwell K. J., Hamilton R. S., Williams S. R. (1997). J. Emerg. Med..

[cit41] Kareem M. A., Mjalli F. S., Hashim M. A., Nashef I. M. Al. (2010). J. Chem. Eng. Data.

[cit42] Dai Y., van Spronsen J., Witkamp G.-J., Verpoorte R., Choi Y. H. (2013). Anal. Chim. Acta.

[cit43] Li H., Chang Y., Zhu W., Wang C., Wang C., Yin S., Zhang M., Li H. (2015). Phys. Chem. Chem. Phys..

[cit44] Lawal A., Lawal M. M., Azeez M. A., Ndungu P. (2019). J. Mol. Liq..

[cit45] Wagle V., Deakyne C. A., Baker G. A. (2016). J. Phys. Chem. B.

